# Clinical utility of diagnostic guidelines and putative biomarkers in lymphangioleiomyomatosis

**DOI:** 10.1186/1465-9921-13-34

**Published:** 2012-04-18

**Authors:** William YC Chang, Jennifer L Cane, John D Blakey, Maruti Kumaran, Kate S Pointon, Simon R Johnson

**Affiliations:** 1 Division of Therapeutics and Molecular Medicine, National Centre for Lymphangioleiomyomatosis and Nottingham Respiratory Biomedical Research Unit, D Floor, South Block, Queen’s Medical Centre, University of Nottingham, Nottingham, NG7 2UH, UK; 2 Division of Respiratory Medicine, University of Nottingham and Nottingham Respiratory Biomedical Research Unit, City Hospital, Nottingham, NG5 1 PB, UK; 3 Department of Radiology, Nottingham University Hospitals NHS Trust, Nottingham, NG7 2UH, UK

**Keywords:** VEGF-D, Matrix metalloproteinase, Angiotensin converting enzyme, ERS LAM guidelines

## Abstract

**Background:**

Lymphangioleiomyomatosis is a rare disease occurring almost exclusively in women. Diagnosis often requires surgical biopsy and the clinical course varies between patients with no predictors of progression. We evaluated recent diagnostic guidelines, clinical features and serum biomarkers as diagnostic and prognostic tools.

**Methods:**

Serum vascular endothelial growth factor-D (VEGF-D), angiotensin converting enzyme (ACE), matrix metalloproteinases (MMP) -2 and -9, clinical phenotype, thoracic and abdominal computerised tomography, lung function and quality of life were examined in a cohort of 58 patients. 32 healthy female controls had serum biomarkers measured.

**Results:**

Serum VEGF-D, ACE and total MMP-2 levels were elevated in patients. VEGF-D was the strongest discriminator between patients and controls (median = 1174 vs. 332 pg/ml p < 0.0001 with an area under the receiver operating characteristic curve of 0.967, 95% CI 0.93-1.01). Application of European Respiratory Society criteria allowed a definite diagnosis without biopsy in 69%. Adding VEGF-D measurement to ERS criteria further reduced the need for biopsy by 10%. VEGF-D was associated with lymphatic involvement (p = 0.017) but not the presence of angiomyolipomas.

**Conclusions:**

Combining ERS criteria and serum VEGF-D reduces the need for lung biopsy in LAM. VEGF-D was associated with lymphatic disease but not lung function.

## Background

Lymphangioleiomyomatosis (LAM) is a rare lung disease which almost exclusively affects women and generally presents before the menopause [1,2]. It can occur sporadically or in association with the genetic condition tuberous sclerosis complex (TSC): both sporadic and TSC-LAM are associated with mutations in the TSC genes[3]. Histologically, LAM is characterised by the abnormal growth of atypical, smooth muscle-like LAM cells in the lungs leading to lung cysts, airway obstruction and in many cases, respiratory failure. Obstruction of axial lymphatics by LAM cells can cause lymphadenopathy and chylous collections[4]. In addition, around 60% of patients with sporadic LAM and most patients with TSC-LAM have one or more angiomyolipomas[5][6][7], benign tumours which share a common ancestry with pulmonary LAM cells [3,8-10]. Importantly, high resolution computerised tomography (HRCT) alone is not diagnostic of LAM and patients undergoing transplant for ‘LAM’ have been found to have other diseases after examination of the explanted lungs [11,12]. Lung biopsy is often used to confirm diagnosis, but recent clinical guidelines specify that a definitive diagnosis can be made without lung biopsy in the presence of lung cysts, plus evidence of either angiomyolipomas, chylous collections or TSC (Table 1) [13]. These diagnostic criteria, based on expert opinion, have not yet been formally evaluated. With the advent of disease specific treatments such as sirolimus [14,15] it is increasingly important to make the correct diagnosis. Furthermore, LAM is a variable disease with some patients developing respiratory failure and others remaining stable for many years. It is currently not possible to predict with confidence the likely clinical course at presentation which is unsettling for patients and makes planning treatment difficult [16].

**Table 1:** Summary of the ERS guidelines for the diagnosis of LAM

	
*Definite LAM*	1. Characteristic or compatible lung HRCT **AND** lung biopsy fitting the pathological criteria for LAM **OR**
	2. Characteristic lung HRCT and any of the following:
	· Angiomyolipoma (kidney)
	· Thoracic or abdominal chylous effusion
	· Lymphangioleiomyoma or lymph node involvement by LAM
	· Definite or probable TSC
*Probable LAM*	1. Characteristic HRCT and compatible clinical history **OR**
	2. Compatible HRCT and any of the following:
	· Angiomyolipoma (kidney)
	· Thoracic or abdominal chylous effusion
*Possible LAM*	Characteristic or compatible HRCT

Circulating biomarkers offer a potential non-invasive method for aiding diagnosis, monitoring disease progression and predicting prognosis. Several putative biomarkers have already been identified in LAM. Vascular endothelial growth factor-D (VEGF-D), a ligand for the lymphatic growth-factor receptor VEGFR-3/Flt-4, is elevated in serum of patients with LAM compared with healthy volunteers and patients with other cystic lung diseases. The clinical utility of VEGF-D is not clear at present, however it has been suggested that a serum VEGF-D level of >800 pg/ml in combination with typical cystic changes on HRCT is specific for LAM [17] though a VEGF-D level <800 pg/ml does not exclude the diagnosis [18]. Matrix metalloproteinases (MMPs) are a family of enzymes involved in many processes including extracellular matrix remodelling, angiogenesis, migration and cell signalling [19,20]. Imbalance between MMPs and their inhibitors has been implicated in a variety of pulmonary disorders including LAM and elevated MMP-9, has been observed in the serum of LAM patients [21]. Finally, membrane bound ACE and other elements of the renin-angiotensin system have been identified in LAM cells by immunohistochemistry [22] and we have noted elevated serum angiotensin converting enzyme (ACE) in patients with LAM.

In order to determine if these clinical guidelines and biomarkers can improve clinical care for LAM patients by reducing the need for invasive diagnostic investigations and making more accurate predictions of prognosis, we have compared the clinical utility of serum VEGF-D, MMP-2, MMP-9 and ACE with the recent ERS diagnostic guidelines, health-related quality of life and clinical phenotype in a national cohort of patients with LAM.

## Methods

### Subjects

Patients were all receiving clinical care at the national referral centre for LAM in the UK. Patients were either participating in a clinical trial where baseline data were used, (NCT00989742, MHRA 03057/0032/001-002, EUDRACT 2007-003745-32, NRES 07/H0403/165) or were in an observational cohort with Nottingham Ethics Committee approval (NRES 05/Q2403/187). All patients provided informed consent. Patients were classified as having either definite or probable LAM according to ERS consensus criteria (Table 1).

All were women over the age of 18. Spirometry and transfer factor (TLCO) were measured according to ARTP/BTS standards [23]. Retrospective lung function results were obtained from clinical records. As short term observation of lung function reduces the accuracy of rate of decline estimates for FEV_1_[16], serial data were used only when greater than three years of observation were available. Thoracic and abdominal HRCT were performed for the clinical trial or if indicated clinically. All patients had blood drawn for biomarker analysis. Full blood count, differential white cell count and C-reactive protein (CRP) were also measured to exclude intercurrent infection but were not part of the biomarker analysis. Participants in the clinical trial completed St George’s Respiratory Questionnaires (SGRQ). Thirty two healthy control women over the age of 18 with no prior history of lung disease had blood taken for biomarker analysis (Nottingham University Ethics Committee approval BT A27 08 2009) with informed consent.

### Serum collection and storage

Blood was collected in serum separator tubes, allowed to clot for 30 minutes at 4°C, centrifuged at 4000 G for 10 minutes and serum stored in aliquots at −80°C.

### VEGF-D, MMP-2, MMP-9 and ACE assays

Serum VEGF-D and MMP-2 were measured using Quantikine Human VEGF-D and MMP-2 Immunoassays (R&D Systems, Minneapolis, MN). Serum MMP-9 was measured using the Duoset Human MMP-9 Immunoassay (R&D Systems) with a previously validated serum dilution of 1:1000 [24,25]. Serum ACE was determined by colourimetric assay based on hydrolysis of furylacryloylphenylalanylglycylglycine to furylacryloylphenylalanine and glycylglycine by serum ACE using an Olympus AU 2700 automated analyser (Olympus Diagnostics, Watford, UK).

### Interpretation of radiology

CT scans were examined independently by two consultant radiologists (MK and KSP) blinded to other study data who scored “yes” or “no” to the presence or absence of lymphatic involvement and renal angiomyolipomas. Only a consensus between both radiologists was accepted. Cases where agreement was not reached were omitted from this part of the study.

### Statistical analysis

Statistical analysis was performed using GraphPad Prism 5.02 (GraphPad Software, San Diego, California, USA) and SPSS 16 (SPSS Inc., Chicago, Illinois, USA). Comparison of age and biomarkers between groups was performed using the Mann–Whitney test and correlation between age and biomarkers was examined using Spearman’s rho (statistical significance set at p < 0.05 for a 2-tailed test). Predictive ability of serum biomarkers was assessed by receiver operator characteristic (ROC) curve analysis.

## Results

### Characteristics of study subjects and application of ERS criteria

Fifty eight patients were recruited. According to ERS criteria, 45 had definite and 13 had probable LAM [13]. Eight patients also had TSC. The 13 classified as probable LAM had a clinical history compatible with LAM, lung cysts on CT, but no extra-pulmonary features or evidence of TSC. 33/58 (57%) presented with pneumothorax, 16/58 (28%) with breathlessness alone, four with chylous collections and six incidentally after CT scans performed for unrelated problems. A lung biopsy had been performed for diagnosis in 22/58 patients. One or more angiomyolipomas defined radiologically or after histological analysis were present in 31/58 patients (53%). Patients were slightly older than control subjects (median age 46 yrs, interquartile range (IQR) 16.0 vs. 36, IQR 12.3, p = 0.0003) which was factored into the analysis. Only two controls and one patient had smoked.

Patients were taking standard therapy for LAM including bronchodilators and progesterone. Those taking sirolimus, doxycycline or post lung transplantation were excluded from the study. Only the 45 patients with definite LAM by ERS criteria were included in the comparison of biomarkers in LAM and controls: those with probable LAM were used to determine the utility of VEGF-D for diagnosis.

### Serum VEGF-D is significantly elevated in patients with LAM and is a better diagnostic marker than MMP-2, -9 and ACE

**Figure 1:**
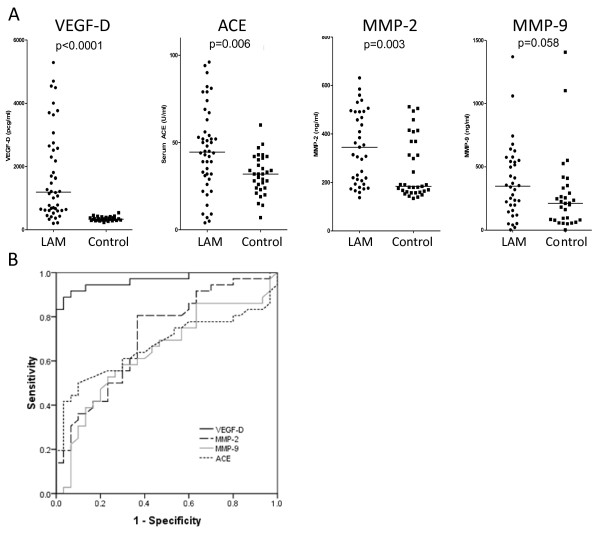
**Biomarker levels in patients with LAM and controls. (a)** Scatter plots comparing patients with definite LAM and controls for: VEGF-D, ACE, MMP-2 and MMP-9. All p values were calculated using Mann–Whitney test and corrected for age. Horizontal lines are the group means. **(b)** Receiver operating characteristic curves for VEGF-D, ACE, MMP-2 and MMP-9.

**Figure 2:**
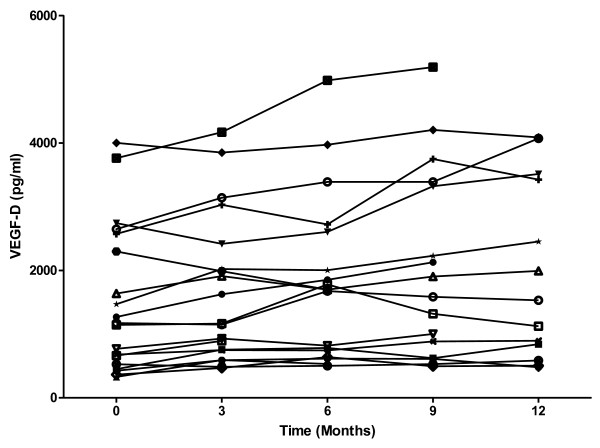
**Stability of VEGF-D over time.** Serum VEGF-D over time for 19 patients with LAM. Each line represents an individual patient.

Spearman correlation performed for age against serum VEGF-D, MMP-2, MMP-9 and ACE showed there was no significant correlation between age and biomarkers with the exception of MMP-2 (p = 0.026). Serum VEGF-D (p < 0.0001), ACE (p = 0.006) and total MMP-2 (p = 0.003) were higher in LAM when compared with controls (Figure 1). There was a trend toward higher total MMP-9 in patients with LAM, although this was not significant (p = 0.058). ROC analysis showed VEGF-D to be the strongest discriminator between those with LAM and controls (area under curve (AUC) 0.967 ± 0.02, 95% CI 0.927-1.007) when compared with ACE (AUC 0.671 ± 0.069, 95% CI 0.538-0.805), MMP-2 (AUC 0.714 ± 0.064, 95% CI 0.590-0.839) and MMP-9 (AUC 0.641 ± 0.069, 95% CI 0.505-0.777) (Figure 1). The sensitivity and specificity of VEGF-D were 56% and 100% respectively with a positive predictive value of 100% and a negative predictive value of 61% using a cut off of 800 pg/ml. In our cohort, a value of 440 pg/ml was the optimal discriminative point, correctly assigning all but 4 cases and all but 1 control. Of the patients with definite LAM by current ERS criteria, 42% had a VEGF-D of less than 800 pg/ml. To examine if VEGF-D was stable over time, we examined serum VEGF-D over a one year period. The mean baseline level was 1522 pg/ml (SD 1156). For the three month period where most data were available, there was a 10% rise in VEGF-D (n = 19 patients, mean rise 158 pg/ml, SD 254). Over this period, none with raised VEGF-D fell below 800 pg/ml but one patient with a borderline level at baseline increased above the 800 pg/ml diagnostic threshold (Figure 2).

### Utility of the ERS criteria combined with VEGF-D for the identification of LAM in patients with cystic lung disease

**Figure 3:**
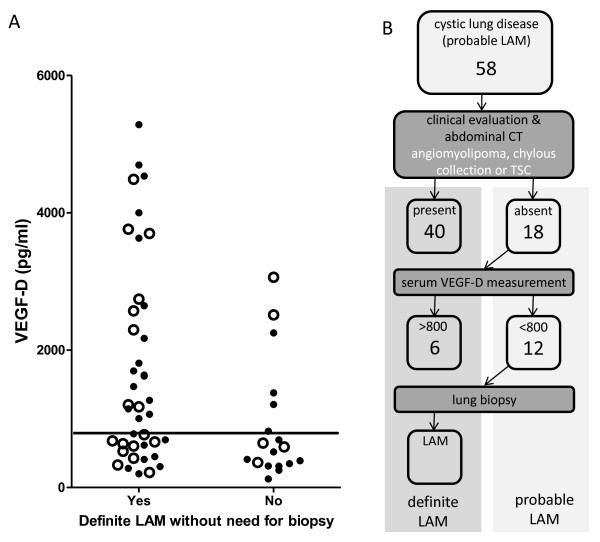
**Analysis of diagnostic utility of VEGF-D.(a)** Patients are classified based on whether a definite diagnosis of LAM using ERS criteria can be made without reference to biopsy data. Open circles indicate those patients who underwent lung biopsy all of whom were confirmed to have LAM by histological analysis. Solid line shows the proposed cut off of 800 pg/ml VEGF-D used to support the diagnosis of LAM. **(b)** Algorithm using diagnostic strategy based upon application of ERS criteria with the inclusion of serum VEGF-D measurement prior to lung biopsy. Figure shows the number from the initial 58 patients in whom a definite diagnosis of LAM can be made at each stage.

As VEGF-D was the most sensitive and specific diagnostic marker we examined whether measurement of serum VEGF-D could reduce the need for lung biopsy in patients with suspected LAM and how VEGF-D measurement contributed to diagnosis as defined by the recent ERS criteria. To do this, patients were reclassified into either ‘definite’ or ‘probable’ LAM according to ERS criteria but without reference to information from lung biopsy. Application of ERS criteria, without biopsy information, allowed a definite diagnosis of LAM in 40 of 58 patients (69%) without the need for biopsy. Interestingly 17 of these 40 had had a lung biopsy, all of which confirmed LAM. Of the remaining 18 patients where ERS criteria could not make a definite diagnosis without lung biopsy, six of these had a VEGF-D level of >800 pg/ml suggesting a further 10% of patients could avoid lung biopsy for a definite diagnosis of LAM were this criteria to be added to diagnostic guidelines (Figure 3).

### Association of serum biomarkers with lung function parameters

**Figure 4:**
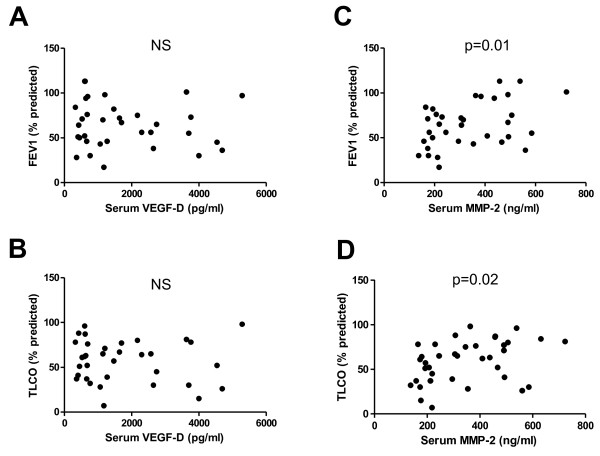
**Correlation of biomarkers with lung function.** VEGF-D levels are not correlated with **(a)** percent predicted FEV_1_ or **(b)** percent predicted TLCO. **(c)** serum MMP-2 levels are associated with percent predicted FEV_1_ and **(d)** percent predicted TLCO.

We next examined if these biomarkers were related to disease severity. VEGF-D, ACE and MMP-9 levels were not associated with percent predicted or absolute FEV_1_ or TLCO. Surprisingly, a higher total MMP-2 was associated with both better FEV_1_ (p = 0.01) and TLCO (p = 0.02) using Spearman 2 tailed correlation whether actual or percent predicted values were considered (Figure 4. ACE and MMP-9 data not shown). A generalized linear model with FEV_1_ percent predicted as the dependent variable and age and biomarkers as covariates confirmed the association with MMP-2 (p = 0.0023), within the limits of our relatively small sample size.

To determine if VEGF-D is associated with disease activity rather than extent of lung function abnormality at a single time point we tested if those with higher VEGF-D experienced a more rapid decline in FEV_1_. Historical lung function data of greater than three years duration were available for 19 patients. The overall rate of decline in FEV_1_ calculated by linear regression was 113 ml/year (range 28–461 ml/year). Rate of FEV_1_ decline in individuals was not associated with VEGF-D level (p = 0.48, Spearman’s Rho).

### VEGF-D is a marker for lymphatic involvement but not angiomyolipomas

**Figure 5:**
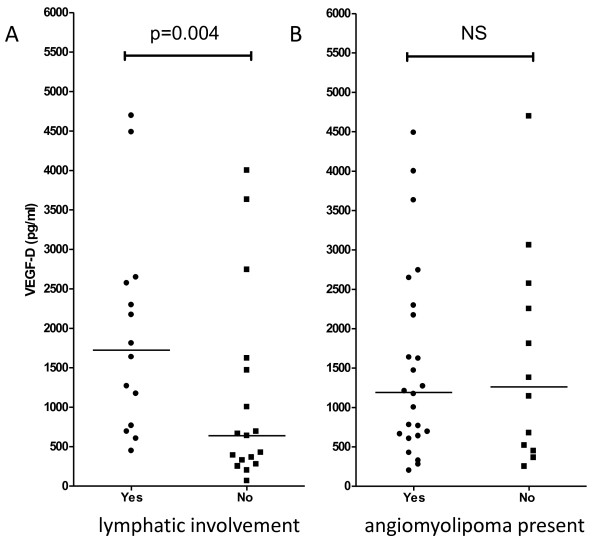
**Association of VEGF-D with lymphatic involvement and angiomyolipomas.** VEGF-D level is associated with **(a)** lymphatic involvement but not **(b)** the presence of angiomyolipomas. (Mann–Whitney test).

As VEGF-D is a lymphangiogenic protein we examined if patients with lymphatic involvement had higher levels of VEGF-D than other patients. In the 29 patients for whom abdominal CT data were available, those with lymphatic involvement had higher VEGF-D levels than those who did not (p = 0.004). VEGF-D was not associated with the presence of angiomyolipomas (Figure 5).

### Quality of life is associated with lung function

**Figure 6:**
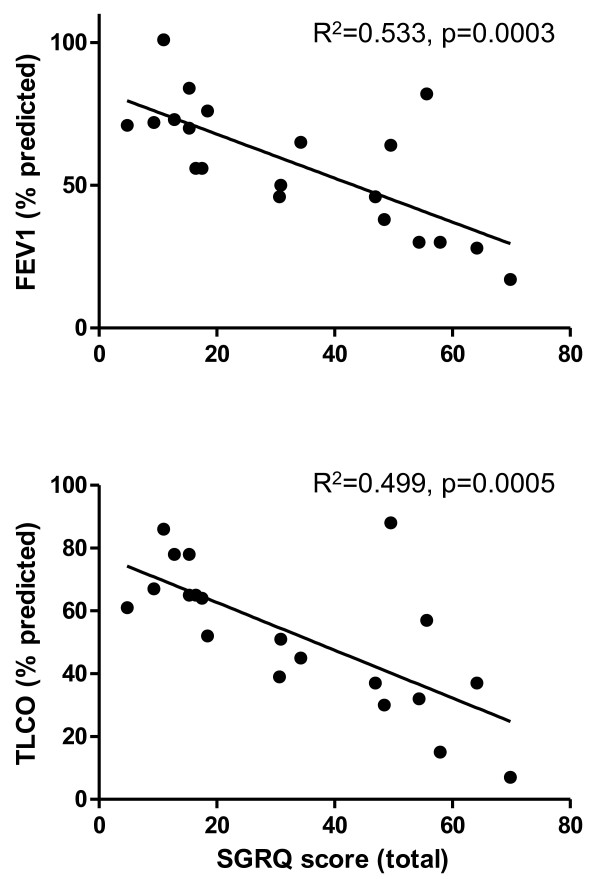
**Correlation of total St. George’s Respiratory Questionnaire score with percent predicted FEV**_**1**_**and percent predicted TLCO.**

Median overall score for the SGRQ was 30.86 (IQR 36.21) and higher overall score was associated with lower percent predicted FEV_1_ (R^2^ = 0.533, p = 0.0003) and TLCO (R^2^ = 0.499, p = 0.0005) (Figure 6). Similar findings were seen with the component scores of symptoms, activity and impact (data not shown).

## Discussion

In this study we evaluated the diagnostic utility of the recent ERS LAM guidelines and clinical use of four serum biomarkers. After prospective application of the ERS criteria to a national cohort, only 30% of these patients remained without a definite diagnosis and this could be reduced to 20% by adding a VEGF-D level of >800 pg/ml. Our data confirm the finding that VEGF-D is elevated in LAM [18,26] and in our cohort, has a diagnostic specificity of 100% but a sensitivity of only 56%: suggesting that an elevated VEGF-D is helpful in confirming the diagnosis of LAM, but a normal VEGF-D level is not sufficient to exclude it. We also show that VEGF-D levels are relatively constant in individuals over time, which improves confidence in the measurement. Although not evaluated here, importantly, *Young et al*. [17] have demonstrated that VEGF-D can also discriminate between LAM and the other cystic diseases, pulmonary Langerhans cell histiocytosis, Birt Hogg Dubé disease and emphysema. The value that best classified subjects into either the LAM or healthy control group was 440 pg/ml in this cohort, this value is lower than that reported by *Young et al.,* probably due to the use of healthy women as our control population. Taken together, our findings suggest that increased knowledge and application of the ERS diagnostic criteria amongst respiratory physicians could reduce the use of lung biopsy in suspected LAM and this could be improved further by incorporating VEGF-D measurement into the diagnostic workup. The observation that VEGF-D is associated with lymphatic disease rather than lung function decline or other aspects of disease activity or phenotype is consistent with a recent clinical trial where VEGF-D in patients with LAM was suppressed by inhibition of the mTOR pathway but did not parallel changes in lung function [15].

In contrast to *Odajima et al.*[21] we found that serum MMP-2 is significantly elevated in LAM. The finding of raised MMP-2 in LAM patient serum is consistent with the observation that LAM derived cells in culture produce excess amounts of MMP-2 which may have therapeutic implications if the protein is activated locally in LAM lesions [27,28]. It is unclear why better lung function is associated with a higher MMP-2 level, although it is unlikely to be an artefact as this was consistent for both FEV_1_ and TLCO. It is possible that MMP-2 production may fall in advanced disease or conversely, MMP-2 may be a protective mechanism against lung destruction. Although MMP-2 and −9 were not useful as diagnostic or prognostic indicators, it remains to be seen whether MMP inhibition will affect disease progression in LAM and the role of MMP-2 requires particular investigation.

The presence of angiotensinogen, angiotensin II, receptors for angiotensin II and ACE in LAM lung tissue has been described by *Valencia et al.*[22] and it has been postulated that a LAM specific renin-angiotensin system may play a role in LAM cell proliferation and migration. Our observation that serum ACE is increased in LAM would be consistent with several possibilities, including release of ACE from LAM cells, cleavage of membrane bound ACE, again, possibly by MMPs [29], or a result of secondary changes to the cardiovascular system in response to the burden of cystic lung disease. The relatively poor separation of ACE levels between patients and controls make this measurement unhelpful as a diagnostic tool.

The overall composite score of the SGRQ and the components relating to symptoms, activity and impact correlated well with percent predicted FEV_1_ and TLCO in keeping with other studies [2,30]. It remains to be seen whether the SGRQ will add any further information above lung function measurements but it deserves evaluation as a marker of treatment response in clinical trials.

Certain caveats apply to the study. Despite this being a relatively large and detailed study for a rare disease, absolute numbers are small for some aspects of the analysis. Also the lung function decline data is retrospective and needs to be repeated in a prospective study. Our patient group encompassed approximately one third of all patients within the country and included the full spectrum of patients from those with no symptoms to those being evaluated for pulmonary transplantation. The cohort is similar to other cohorts with respect to age at presentation and rate of decline in lung function and we feel this makes our findings applicable to a range of patients being evaluated for possible LAM.

## Conclusions

Of the biomarkers examined, serum VEGF-D alone had a sufficient positive predictive value for use as a diagnostic test for LAM. When used in combination with the ERS diagnostic criteria 80% of patients could avoid lung biopsy for diagnosis. Total MMP-2 was elevated in LAM patients although the significance of these observations needs further investigation at a mechanistic level. With the exception of total MMP-2, no serum biomarker was associated with lung function or disease activity. The SGRQ correlates well with FEV_1_ and TLCO in patients with LAM and may be a useful assessment tool, particularly in those who find pulmonary function tests difficult. Its role as an endpoint in an interventional study should be evaluated.

## Funding

The study was funded by the British Lung Foundation, LAM Action and the Nottingham Respiratory Biomedical Research Unit.

## Author contribution

WYCC recruited patients, performed some assays, analysed the data and wrote some of the manuscript. JLC contributed to the biomarker measurements and data analysis, MK and KSP evaluated all radiology and contributed to data analysis, JB performed the statistical modelling and helped with data analysis. SRJ conceived and designed the study, participated in its coordination, data analysis and co-wrote the manuscript. All authors have read and approved the final manuscript.
